# Flow cytometry allows rapid detection of protein aggregates in cellular and zebrafish models of spinocerebellar ataxia 3

**DOI:** 10.1242/dmm.049023

**Published:** 2021-10-11

**Authors:** Katherine J. Robinson, Madelaine C. Tym, Alison Hogan, Maxinne Watchon, Kristy C. Yuan, Stuart K. Plenderleith, Emily K. Don, Angela S. Laird

**Affiliations:** Centre for Motor Neuron Disease Research, Department of Biomedical Sciences, Faculty of Medicine, Health and Human Sciences, Macquarie University, Sydney, NSW 2109, Australia

**Keywords:** Spinocerebellar ataxia 3, Machado–Joseph disease, Hereditary spinocerebellar ataxias, Neurodegenerative disease, Flow cytometry, Proteinopathy, Insoluble protein species, Protein aggregates

## Abstract

Spinocerebellar ataxia 3 (SCA3, also known as Machado–Joseph disease) is a neurodegenerative disease caused by inheritance of a CAG repeat expansion within the *ATXN3* gene, resulting in polyglutamine (polyQ) repeat expansion within the ataxin-3 protein. In this study, we have identified protein aggregates in both neuronal-like (SHSY5Y) cells and transgenic zebrafish expressing human ataxin-3 with expanded polyQ. We have adapted a previously reported flow cytometry methodology named flow cytometric analysis of inclusions and trafficking, allowing rapid quantification of detergent insoluble forms of ataxin-3 fused to a GFP in SHSY5Y cells and cells dissociated from the zebrafish larvae. Flow cytometric analysis revealed an increased number of detergent-insoluble ataxin-3 particles per nuclei in cells and in zebrafish expressing polyQ-expanded ataxin-3 compared to those expressing wild-type human ataxin-3. Treatment with compounds known to modulate autophagic activity altered the number of detergent-insoluble ataxin-3 particles in cells and zebrafish expressing mutant human ataxin-3. We conclude that flow cytometry can be harnessed to rapidly count ataxin-3 aggregates, both *in vitro* and *in vivo*, and can be used to compare potential therapies targeting protein aggregates.

This article has an associated First Person interview with the first author of the paper.

## Abstract (precis)

**Highlighted Article:**Description of a flow cytometric methodology for the rapid detection and quantification of detergent-insoluble protein aggregates within cultured cells and transgenic zebrafish modelling spinocerebellar ataxia 3.

## INTRODUCTION

Spinocerebellar ataxia 3 [SCA3, also known as Machado–Joseph disease (MJD)] is a devastating neurodegenerative disease that causes progressive ataxia (loss of balance and co-ordination) and paralysis, as well as impaired speech, swallowing and vision due to the progressive death of neurons in the central nervous system (Costa and Paulson, 2012). SCA3 is the most common form of hereditary ataxia found throughout the world (21-28% of autosomal-dominant ataxia; Durr et al., 1996; Ranum et al., 1995; Schöls et al., 1995), affecting ∼1-5 people per 100,000 worldwide.

SCA3 is caused by inheritance of an abnormal form of a gene called *ATXN3* (Kawaguchi et al., 1994; Takiyama et al., 1993). The *ATXN3* gene normally contains a repetitious sequence of genetic code (CAG repeat region), which in turn encodes a long string of glutamine (Q) residues (known as a polyQ region) within the ataxin-3 protein. The normal (wild-type) form of the ataxin-3 protein typically contains 12-44 glutamine residues within the polyQ region. Expression of ataxin-3 with more than 44 glutamine residues produces SCA3 disease in patients (Costa and Paulson, 2012; Maciel et al., 1995). A direct relationship exists between the length of this polyQ region and the severity of SCA3, with patients carrying longer repeat lengths suffering earlier disease onset, more severe disease and earlier death (Maciel et al., 1995; Jardim et al., 2001; Abe et al., 1998; Kieling et al., 2007).

Neuropathological staining of SCA3 patient brain samples often reveals the presence of ataxin-3^+^ neuronal intranuclear inclusions (Schmidt et al., 1998). Formation of these ataxin-3 protein aggregates and inclusions is hypothesized to play a role in the neuronal dysfunction and degeneration that occurs in SCA3 (Schmidt et al., 1998; Rüb et al., 2013; Yamada et al., 2000). Although the ataxin-3 protein has been found to function as a de-ubiquitinating enzyme (Scheel et al., 2003), it has also been found to sequester other essential proteins into protein aggregates, altering the function of sequestered proteins (Yang et al., 2014; Donaldson et al., 2003). Similarly, other proteins containing polyglutamine tracts, such as huntingtin and the androgen receptor, have also been found to aggregate when the polyglutamine tract is expanded, resulting in neurodegenerative diseases, such as Huntington's disease and Kennedy's disease, respectively (Gusella and MacDonald, 2000; Hands and Wyttenbach, 2010). For these reasons, approaches to identify therapeutics that can prevent, or reverse, protein aggregation processes are currently an area of thorough investigation within the field (Rubinsztein et al., 2015).

Whiten et al. (2016) previously established a flow cytometric approach enabling rapid enumeration of insoluble protein inclusions per nuclei present in cell culture models, coined flow cytometric analysis of inclusions and trafficking (FloIT). This approach has since been used to rapidly quantify the number of detergent insoluble inclusions across many *in vitro* models expressing aggregation-prone proteins, including mutant huntingtin (linked with Huntington's disease) (Whiten et al., 2016) and motor neuron disease-linked proteins SOD1 (Whiten et al., 2016; McAlary et al., 2016) and TDP-43 (Zeineddine et al., 2017a,b). The application of FloIT has also been expanded to investigate other cellular processes associated with protein aggregation, including induction of a heat shock response (San Gil et al., 2020), the role of molecular chaperones in suppressing protein aggregation (McMahon et al., 2021) and chaperone-assisted selective autophagy (Adriaenssens et al., 2020). Considering that the formation of detergent-insoluble protein aggregates is a disease mechanism common to a wide variety of diseases, including Alzheimer's disease, Parkinson's disease, polyglutamine diseases, such as Huntington's disease and spinocerebellar ataxias, and even type 2 diabetes and dilated cardiomyopathy (Adriaenssens et al., 2020; Chiti and Dobson, 2017; Ross and Poirier, 2004; Taylor et al., 2002; Gidalevitz et al., 2006; Mukherjee et al., 2015), it is important to determine whether FloIT can be widely adopted to study other proteinopathy disease models. This is especially important to consider seeing as different proteinopathy diseases are characterised by the formation of protein aggregates in different subcellular compartments. For example, protein aggregates found in models of motor neuron disease and Huntington's disease are more commonly present within the cell cytoplasm, whereas in other diseases, such as forms of spinocerebellar ataxia, protein aggregates can also be detected within the cell nucleus.

In the present study, we demonstrate that FloIT can be adapted to quantify the number and size of detergent-insoluble enhanced GFP (EGFP)-fused ataxin-3 particles in neuronal-like (SHSY5Y) cells transiently expressing ataxin-3 containing a short polyQ repeat length (28 glutamine residues) or a long polyQ repeat length (84 glutamine residues). We also report an adaption of this methodology for use with our previously reported transgenic zebrafish model of SCA3 (Watchon et al., 2017). This adaption is important because zebrafish are an excellent model for moderate to high throughput assessment of drug efficacy, as overexpression of human genes can produce disease phenotypes akin to human disease phenotypes, including impaired movement, within days. Further, potential therapeutic compounds can be diluted into the water the fish live in and are easily absorbed (Zon and Peterson, 2005). This is the first report of the use of such an approach with an *in vivo* model and provides a novel tool for assessing efficacy in drug testing studies investigating treatments for proteinopathy diseases.

## RESULTS

### Expression of human ataxin-3 with an expanded polyglutamine region in neuronal-like SHSY5Y cells results in the formation of EGFP^+^ aggregates

**Fig. 1.**
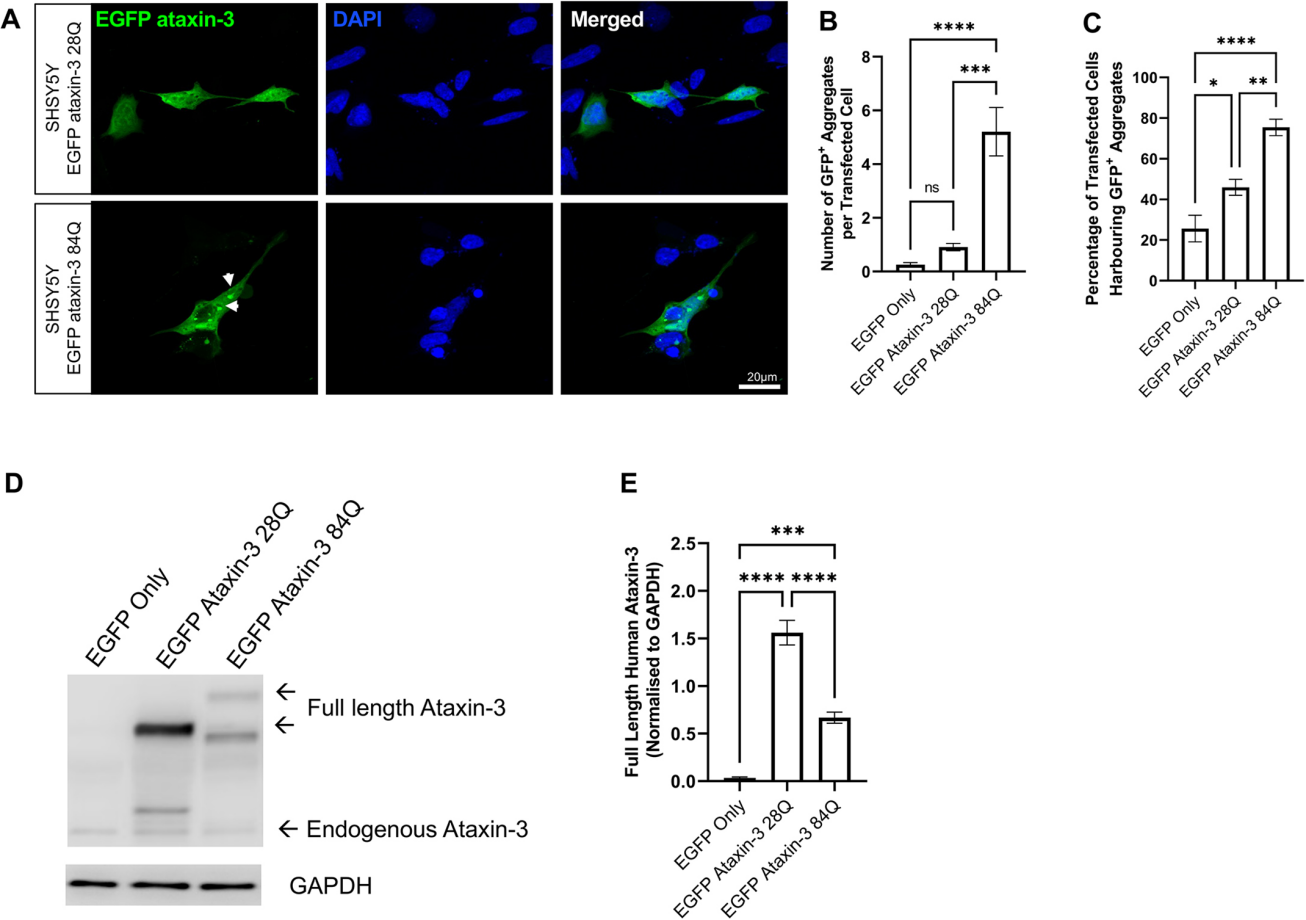
**Neuronal-like SHSY5Y cells develop EGFP ataxin-3 protein aggregates.** (A) SHSY5Y cells transiently expressing EGFP-fused human ataxin-3 (28Q and 84Q) develop protein aggregates. White arrowheads indicate EGFP-fused ataxin-3 protein aggregates. (B) The mean number of aggregates per transfected cell was calculated by averaging six experimental replicates (different fields of view) per coverslip. Aggregates were more commonly observed in cells transfected with the EGFP ataxin-3 84Q repeat expansion than cells transfected with EGFP ataxin-3 28Q repeat expansion. (C) Quantification of the percentage of cells harbouring protein aggregates revealed significantly more cells were affected by protein aggregates following transient transfection with EGFP ataxin-3 84Q compared to EGFP ataxin-3 28Q or EGFP only. (D) Western blotting was used to examine the expression of ataxin-3 following transient transfection of EGFP constructs. (E) Expression of EGFP-fused ataxin-3 was significantly higher in cells transfected with EGFP only or EGFP ataxin-3 84Q. Data are mean±s.e.m. *n*=4-5 experimental replicates. *P<0.05; **P<0.01; ***P<0.001; ****P<0.0001; ns, not significant (one-way ANOVA with Tukey's post-hoc test).

Microscopic analysis of SHSY5Y cells expressing EGFP fused-human ataxin-3 containing 28Q (EGFP ataxin-3 28Q), EGFP ataxin-3 84Q or EGFP alone revealed the presence of more frequent EGFP^+^ aggregates within the cells expressing human ataxin-3 containing 84Q than those expressing 28Q or EGFP-only vector control (Fig. 1A). Manual counting of the number of EGFP^+^ protein aggregates and one-way ANOVA revealed a significant difference in the number of EGFP^+^ aggregates across genotypes (*P*<0.0001). Post-hoc comparisons revealed that more aggregates were present in SHSY5Y cells expressing EGFP ataxin-3 84Q compared to cells expressing EGFP alone (*P*<0.0001) or EGFP ataxin-3 28Q (*P*=0.0002; Fig. 1B). Further, analysis of manual counts revealed a difference in the number of cells affected with protein aggregates across genotypes (*P*<0.0001, Fig. 1C). Multiple comparisons revealed that cells transiently expressing EGFP-fused ataxin-3 84Q displayed a significantly higher percentage of cells harbouring EGFP^+^ aggregates than cells expressing EGFP only (*P*<0.0001) and cells expressing EGFP-fused ataxin-3 with 28Q (*P*=0.0033).

Next, we used western blotting to examine the relative expression of ataxin-3 across our three cell culture expression models, as the relative amount of protein expressed could influence ataxin-3 aggregation. Immunoblot analysis of SHSY5Y cells transiently expressing EGFP-fused human ataxin-3 or an EGFP only control revealed expression of endogenous ataxin-3 and overexpression of EGFP-fused human ataxin-3 (Fig. 1D, see Fig. S2A for full immunoblot). The expression of EGFP-fused human ataxin-3 differed across the examined genotypes (*P*<0.0001, Fig. 1E), with expression of human ataxin-3 higher in cells expressing EGFP-fused ataxin-3 28Q and EGFP-fused ataxin-3 84Q compared to cells expressing EGFP only (*P*<0.0001 and *P*=0.0001, respectively). Interestingly, the expression of EGFP-fused ataxin-3 28Q was significantly increased compared to the expression of EGFP-fused ataxin-3 84Q (*P*<0.0001), suggesting the increased presence of ataxin-3 aggregates in our SCA3 cells is not due to underlying differences in protein expression.

**Fig. 2.**
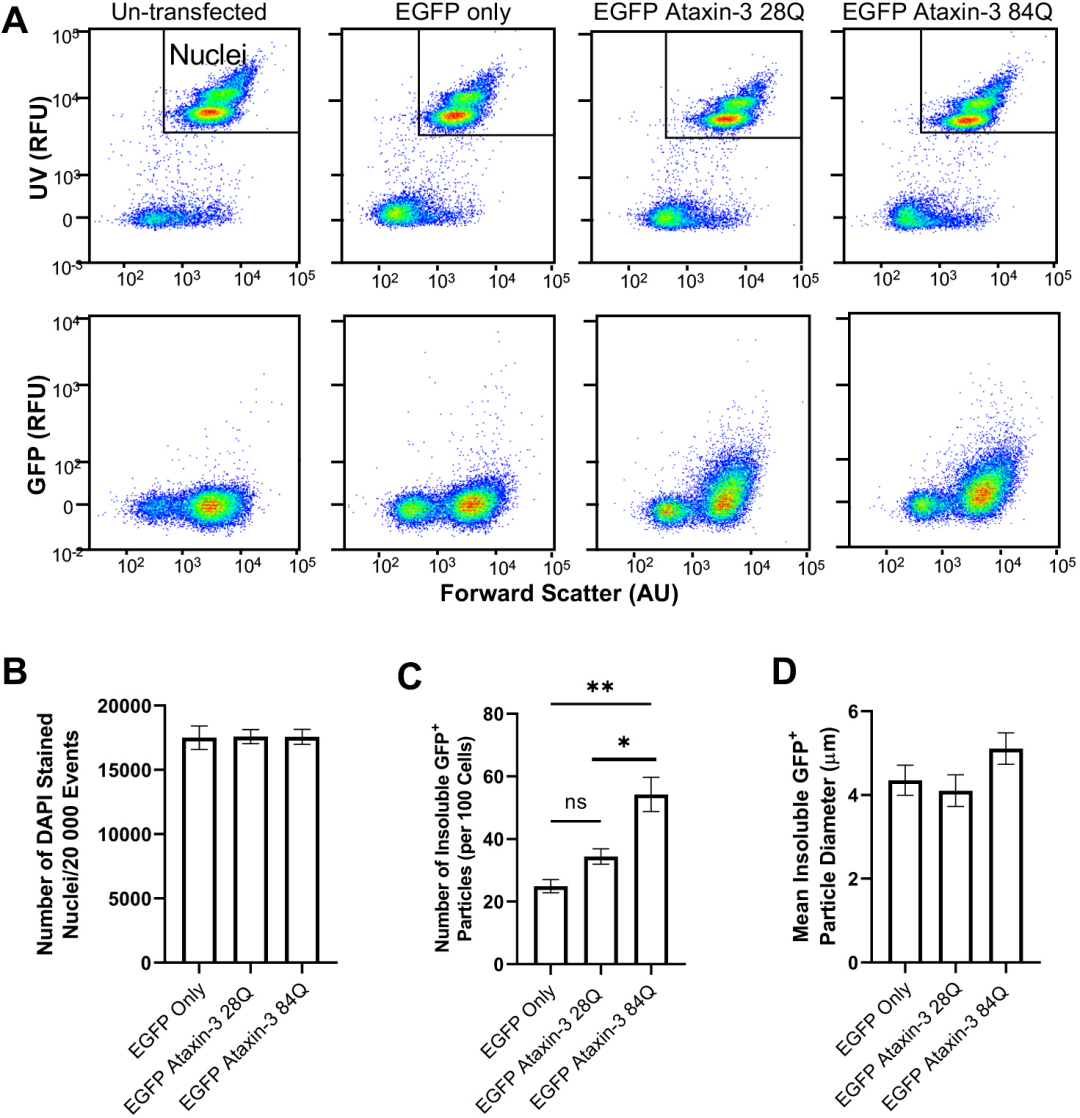
**Flow cytometry can be used to quantify the number of detergent-insoluble EGFP ataxin-3 particles relative to the number of nuclei.** (A) Representative flow cytometry scatterplots displaying UV^+^ nuclei and EGFP-fused ataxin-3 particles from cell lysates. (B) Quantification of the number of DAPI^+^ nuclei revealed that similar numbers of nuclei were present in the analysed samples. (C) Cells expressing EGFP-fused mutant ataxin-3 (84 glutamines) developed significantly more insoluble particles compared to EGFP alone or EGFP-fused ataxin-3 with a short polyglutamine stretch (28 glutamines). (D) Detergent-insoluble particle size did not significantly differ across the examined genotypes. Data are mean±s.e.m. *n*=4-9 experimental replicates. *P<0.05; **P<0.01; ns, not significant (one-way ANOVA with Tukey's post-hoc test). AU, arbitrary units; RFU, relative fluorescence units.

We next validated the rapid quantification of the number of Triton X-100 insoluble aggregates using the FloIT methodology reported previously (Whiten et al., 2016). Transfected cells underwent flow cytometric analysis to quantify the number and size of Triton X-100 insoluble particles (Fig. 2A). First, fluorescent microscopy was used to confirm the number of transfected cells per sample (prior to harvesting). The calculated transfection efficiency of each sample was used to calculate the total number of detergent-insoluble particles per sample. Next, the total number of nuclei present within the lysed sample was determined by staining for DAPI. The number of DAPI^+^ particles was quantified using population gating based on the intensity of UV fluorescence and relative size (forward scatter). No statistically significant differences in the number of DAPI^+^ nuclei were detected across the examined groups (*P*=0.996, Fig. 2B). The number of Triton X-100 insoluble EGFP^+^ particles was then identified via gating of particle populations present within cells expressing EGFP constructs but absent in the untransfected control sample (Whiten et al., 2016). The number of Triton X-100 insoluble EGFP^+^ particles was calculated using the FloIT equation: 100×(number of insoluble GFP particles/number of DAPI^+^ nuclei×transfection efficiency), as reported previously by Whiten et al. (2016). This equation revealed a statistically significant difference across the analysed genotypes (one-way ANOVA, *P*=0.0013, *n*=4-9 experimental replicates per group), with a greater number of detergent-insoluble particles per 100 cells found in EGFP ataxin-3 84Q-expressing SHSY5Y cells compared to cells expressing EGFP alone (*P*=0.0022) and EGFP ataxin-3 28Q (*P*=0.0118) (Fig. 2C). No significant differences were found between the number of detergent-insoluble particles expressed in EGFP alone or in EGFP ataxin-3 28Q-expressing cells (*P=*0.4328). Additionally, the use of non-fluorescent microspheres of known size allowed us to identify that EGFP ataxin-3 84Q cells harboured detergent-insoluble particles that were similar in size to cells expressing EGFP ataxin-3 28Q and EGFP alone (Fig. 2D).

### Treatment of SCA3 cell cultures with compounds known to modulate autophagic activity altered the number of EGFP-fused ataxin-3 particles detected by FloIT

**Fig. 3.**
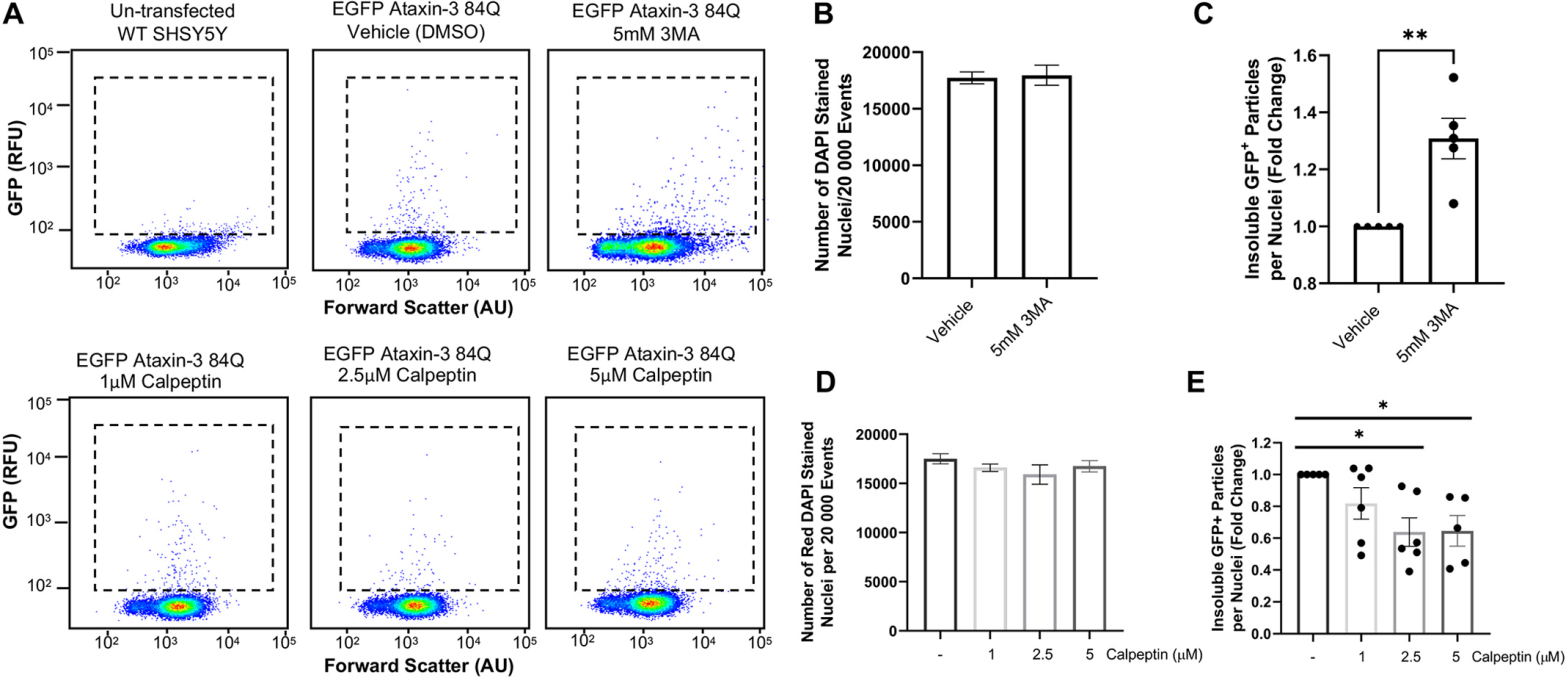
**Treatment with autophagy pathway modulators altered the number of EGFP-fused particles present in cells expressing human ataxin-3 with 84 glutamines.** (A) Representative scatterplots reveal a scarcity of EGFP-fused particles in untransfected control cells and altered numbers of particles following treatment with autophagy modulators (3MA and calpeptin). (B) Quantification of the number of DAPI^+^ nuclei revealed that a similar number of nuclei were present across vehicle-treated and 3MA-treated samples. (C) Comparison of cells treated with vehicle or 3MA (5 mM) revealed a statistically significant increase in detergent-insoluble particles relative to the number of nuclei following 3MA treatment. (D) Similar numbers of nuclei were quantified in samples treated with a vehicle control or calpeptin. (E) Calpeptin treatment produced reductions in detergent-insoluble particles, as detected by FloIT. Data are mean±s.e.m. *n*=2-6 experimental replicates. *P<0.05, **P<0.01 (one-way ANOVA with Tukey's post-hoc test or Student's *t*-test, paired, two-tailed). AU, arbitrary units; RFU, relative fluorescence units.

To validate the use of FloIT as a tool to screen the effect of compounds on detergent-insoluble ataxin-3 protein species, we treated cells transiently transfected with EGFP ataxin-3 84Q for 24 h with 3-methyladenine (3MA), a known inhibitor of autophagy (Klionsky et al., 2021; Seglen and Gordon, 1982), and calpeptin, a compound known to induce autophagy and reduce proteinopathy in polyQ disease models (Watchon et al., 2017; Haacke et al., 2007; Menzies et al., 2015). Indeed, we found that 24-h treatment with modulators of the autophagy protein quality control pathway altered the number of EGFP particles detected by flow cytometry (Fig. 3A). We found that treatment with 3MA did not alter the number of DAPI^+^ nuclei detected within each sample (*P=*0.838, Fig. 3B). In contrast, treatment with 3MA produced a 1.3-fold increase in the number of detergent-insoluble EGFP particles present within cells expressing EGFP ataxin-3 84Q compared to vehicle treatment (*P*=0.002, Fig. 3C). Next, we examined the effect of increasing doses of calpeptin on the number of Triton X-100 insoluble particles detected in cell culture samples transfected with EGFP ataxin-3 84Q. Calpeptin treatment did not alter the number of nuclei detected within each sample (*P*=0.438, Fig. 3D); however, calpeptin did alter the number of detergent-insoluble EGFP particles detected (*P*=0.028, Fig. 3E). Post-hoc comparisons revealed that treatment with 1 μM calpeptin did not significantly alter the number of insoluble particles detected compared to vehicle treatment (*P*=0.331). In contrast, treatment with 2.5 μM or 5 μM calpeptin produced a statistically significant decrease in the number of detergent-insoluble particles compared to vehicle treatment (*P*=0.021 and *P*=0.031, respectively).

### Expression of expanded human *ATXN3* in transgenic zebrafish results in the formation of ataxin-3^+^ aggregates

**Fig. 4.**
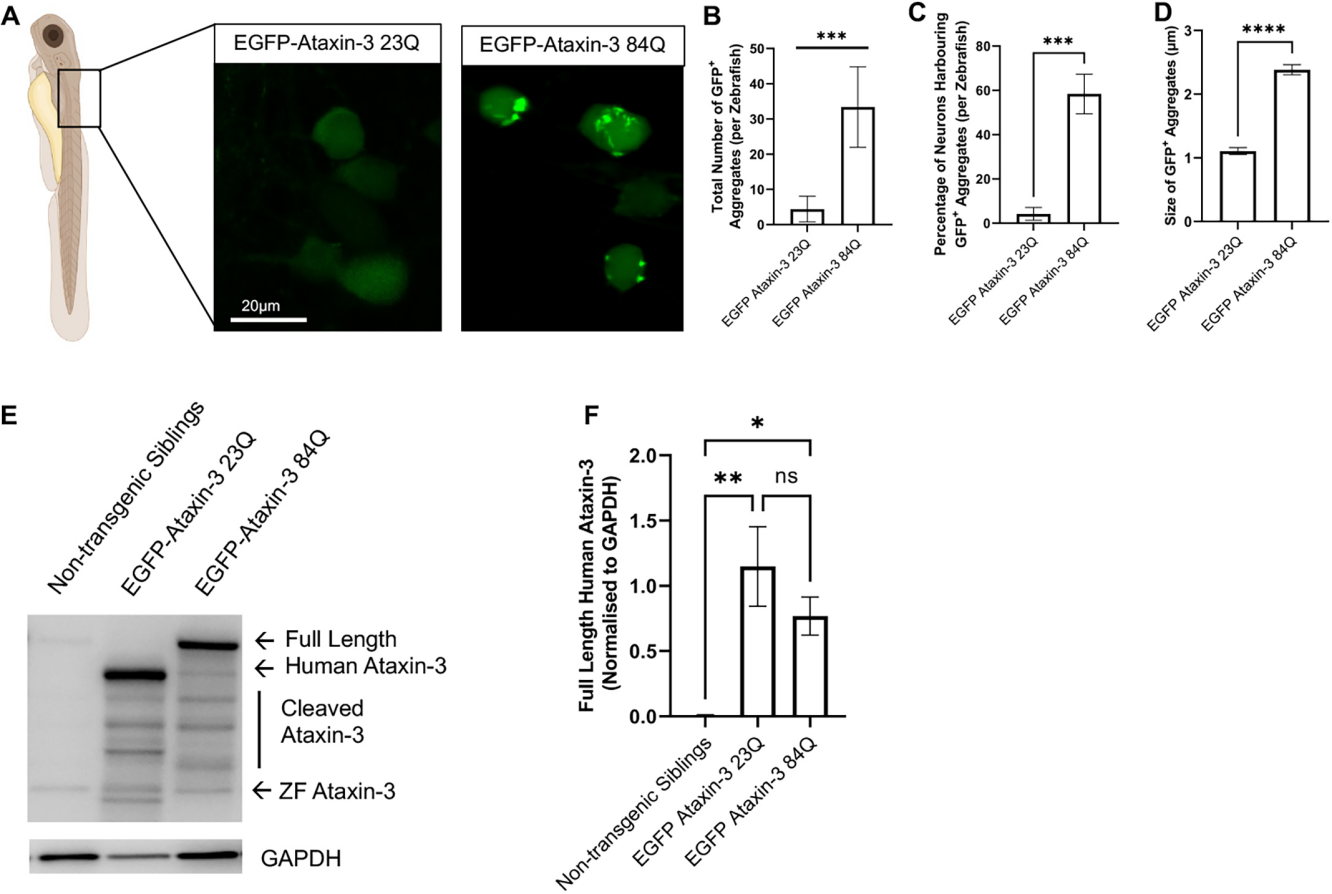
**Transgenic zebrafish expressing human ataxin-3 with 84 glutamines develop ataxin-3^+^ protein aggregates.** (A) Confocal microscopy was performed on 6-day-old zebrafish larvae expressing EGFP-tagged human ataxin-3 containing a short polyglutamine stretch (28Q) or long polyglutamine stretch (84Q). (B) Manual counting of EGFP aggregates revealed that significantly more aggregates were present in 6-dpf zebrafish expressing EGFP ataxin-3 84Q than those expressing EGFP ataxin-3 23Q. (C) Quantification of the percentage of cells harbouring protein aggregates revealed that a greater number of cells were affected with aggregates in zebrafish expressing EGFP ataxin-3 84Q. (D) Measurement of the size of protein aggregates observed via confocal microscopy revealed that zebrafish expressing EGFP ataxin-3 84Q also display aggregates that are significantly larger in size compared to the aggregates found in zebrafish expressing EGFP ataxin-3 23Q. (E) Western blotting was used to examine the relative expression of human ataxin-3 across the examined genotypes. (F) Densiometric analysis revealed significantly greater expression of EGFP-fused human ataxin-3 in transgenic zebrafish compared to non-transgenic siblings. Data are mean±s.e.m. *P<0.05; **P<0.01; ***P<0.001; ****P<0.0001; ns, not significant (one-way ANOVA with Tukey's post-hoc test or Student's *t*-test, paired, two-tailed). *n*=4-5 experimental replicates.

We have previously described a transgenic zebrafish model of SCA3 that overexpresses EGFP-fused human ataxin-3 with an expanded polyQ (84Q) tract, that develops impaired swimming behaviour and shortened lifespan (Watchon et al., 2017). Furthermore, histological analysis of the medulla from adult zebrafish (12 months of age) expressing human mutant ataxin-3 (84Q) evidenced a neuritic beading pattern that was positive for both ataxin-3 and ubiquitin (Watchon et al., 2017). In this current study, we performed confocal microscopy on transgenic zebrafish larvae expressing human ataxin-3 at 6 days post fertilisation (dpf) to examine the rostral spinal cord (Fig. 4A). We observed similar levels of overall human ataxin-3 expression in zebrafish expressing ataxin-3 with 23 glutamine residues and ataxin-3 with 84 glutamine residues, and observed EGFP^+^ protein aggregates in neurons in both transgenic models. Manual counting of the EGFP^+^ protein aggregates revealed a statistically significant increase in the number of aggregates in larvae expressing EGFP ataxin-3 84Q than in those expressing EGFP ataxin-3 23Q (*P*=0.0007; *n*=5 zebrafish larvae imaged per ataxin-3 construct, Fig. 4B). In addition to quantifying the number of aggregates present per zebrafish, we also quantified the percentage of neurons that harboured EGFP^+^ protein aggregates. Transgenic SCA3 zebrafish possessed a significantly higher percentage of cells harbouring ataxin-3 aggregates compared to zebrafish expressing EGFP ataxin-3 23Q (*P*=0.0004, Fig. 4C). Measurement of the size of observed protein aggregates within our maximum intensity microscopy images revealed that transgenic zebrafish expressing EGFP ataxin-3 23Q had significantly smaller aggregates (∼1 μm) compared to zebrafish expressing EGFP ataxin-3 84Q (containing aggregates greater than 2 μm in diameter) (Fig. 4D).

To confirm that the increased presence of ataxin-3 aggregates in transgenic SCA3 zebrafish was not due to underlying differences in protein expression, protein lysates from 2-dpf zebrafish were obtained and immunoblotted to examine ataxin-3 expression. Immunoblotting revealed the presence of endogenous zebrafish ataxin-3, full-length EGFP-fused human ataxin-3 and the presence of cleaved human ataxin-3 species (Fig. 4E, see Fig. S2B for full immunoblot). Densiometric analysis revealed a statistically significant difference in the expression of human ataxin-3 across the examined genotypes (*P*=0.0043, Fig. 4F). Post-hoc comparisons revealed significantly higher expression of human ataxin-3 in transgenic zebrafish expressing EGFP ataxin-3 23Q or EGFP ataxin-3 84Q compared to non-transgenic siblings (*P*=0.0037 and *P*=0.0428, respectively). No statistically significant differences were detected in the expression of human ataxin-3 between EGFP ataxin-3 23Q- and EGFP ataxin-3 84Q-expressing larvae (*P*=0.381). These findings align with our previous reports of similar levels of ataxin-3 in this same transgenic zebrafish line at 3 dpf and 6 dpf (Watchon et al., 2017).

### Flow cytometry can be used to quantify the number of ataxin-3 protein aggregates in transgenic zebrafish

**Fig. 5.**
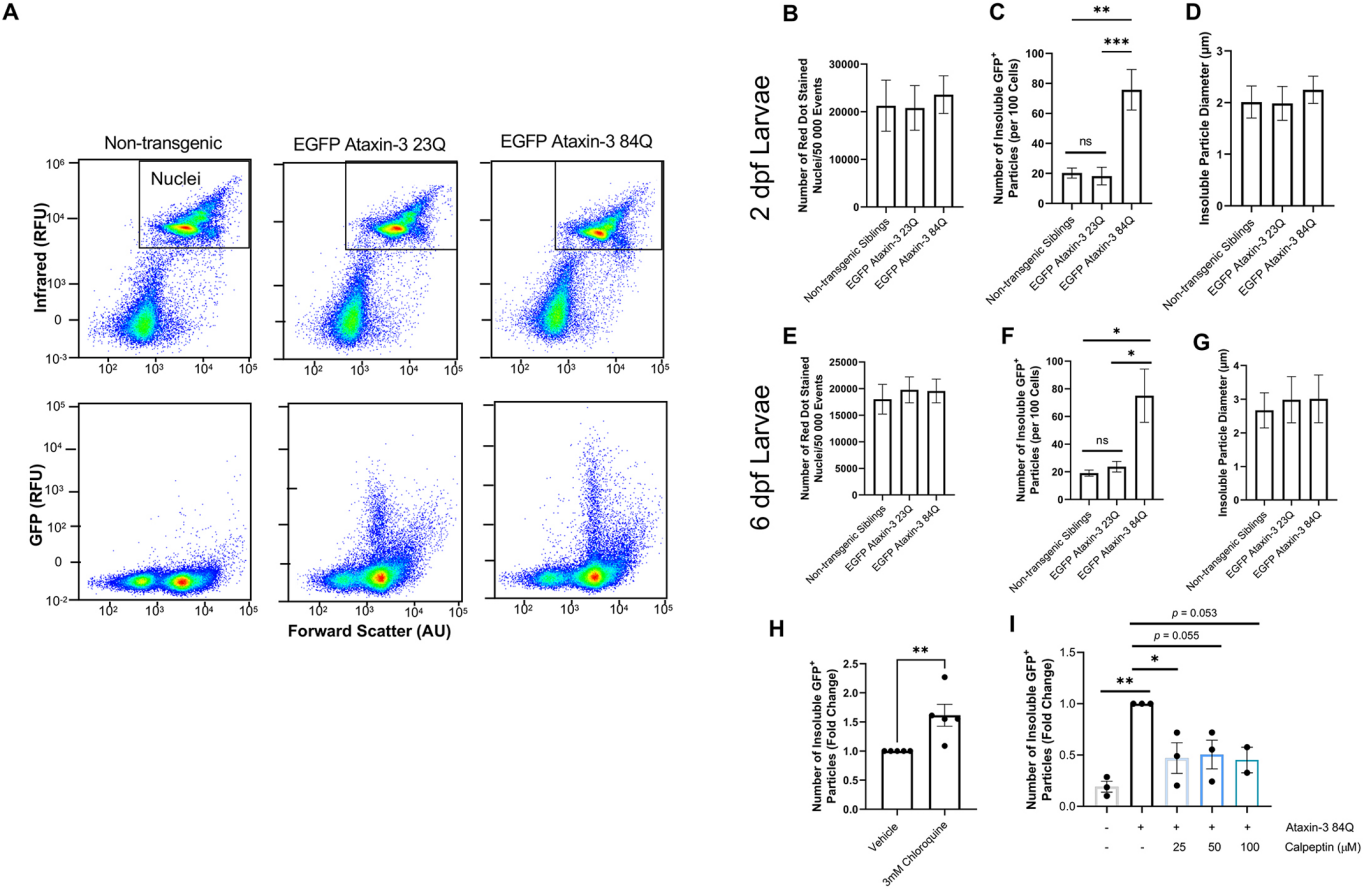
**Flow cytometric analysis of dissociated cells from whole zebrafish can be used to rapidly quantify insoluble EGFP ataxin-3 particles and screen compounds for the effect on proteinopathy.** (A) Representative scatterplots of flow cytometric populations from dissociated 6-day-old zebrafish revealed changes in the number of detected detergent-insoluble particles despite similar numbers of nuclei. (B,C) Zebrafish expressing human mutant ataxin-3 displayed similar numbers of RedDot-stained nuclei but significantly more detergent-insoluble ataxin-3 particles at 2 days of age. (D) Examination of insoluble particle size revealed similar-sized particles across genotypes. (E,F) At 6 dpf, cells from dissociated zebrafish showed similar numbers of nuclei but more EGFP ataxin-3 particles were detected in cells obtained from zebrafish expressing human mutant ataxin-3 compared to non-transgenic siblings and zebrafish larvae expressing human wild-type ataxin-3. (G) Detergent-insoluble particles were a similar size in cells obtained from zebrafish expressing wild-type human ataxin-3 and mutant human ataxin-3. (H) Treatment with the autophagy inhibitor chloroquine (3 mM) increased the number of detergent-insoluble particles detected. (I) Treatment with the autophagy inducer calpeptin decreased the number of detergent-insoluble particles in 2-dpf zebrafish expressing mutant ataxin-3. Data are mean±s.e.m. *n*=2-9 experimental replicates. *P<0.05; **P<0.01; ***P<0.001; ns, not significant (one-way ANOVA with Tukey's post-hoc test or Student's *t*-test, paired, two-tailed). AU, arbitrary units; RFU, relative fluorescence units.

We then modified the FloIT approach to allow quantification of the number of detergent-insoluble EGFP^+^ protein aggregates within cells dissociated from whole zebrafish larvae (2 dpf or 6 dpf) expressing human EGFP-fused ataxin-3. Following euthanasia, manual dissection and trypsinisation of whole zebrafish larvae bodies resulted in a suspension of dissociated cells that could then be lysed with 0.5% Triton X-100, and detergent-insoluble populations were examined using FloIT (Fig. 5A). Visualisation of lysed particles using an infrared laser (excitation 640 nm, emission 670 nm) revealed similar numbers of RedDot-labelled nuclei present within 2-dpf samples (*P*=0.905, Fig. 5B), confirming an equal number of cells per sample. In contrast, visualisation of particles using a blue-green laser (excitation 488 nm, emission 525 nm) revealed the presence of detergent-insoluble EGFP^+^ populations in the cells dissociated from the zebrafish larvae. These EGFP^+^ particles were detectable as early as 2 dpf, at which point one-way ANOVA revealed a significant difference in the number of detergent-insoluble EGFP^+^ particles per 100 cells across the examined genotype groups (*P*=0.0004, Fig. 5C), with significantly more detergent-insoluble EGFP^+^ particles in dissociated zebrafish expressing EGFP ataxin-3 84Q compared to non-transgenic siblings (*P*=0.0012) and EGFP ataxin-3 23Q larvae (*P*=0.0009). Interestingly, the use of calibrated microspheres revealed that the size of the detergent-insoluble particles was similar across the genotypes and consistent with aggregate diameters found in other *in vivo* models of SCA3 (Santana et al., 2020) and SCA3 patient brain tissue (Paulson et al., 1997) (Fig. 5D).

We also examined aggregate number and size at 6 dpf, an age at which the SCA3 transgenic zebrafish present with a swimming phenotype (Watchon et al., 2017). Again, similar numbers of nuclei were detected within 6-dpf samples (*P*=0.861, Fig. 5E). We also found that the number of detergent-insoluble EGFP-fused ataxin-3 aggregates was consistent at both timepoints examined, with a significant difference across genotype groups also evident at 6 dpf (*P*=0.0134). Post-hoc analysis revealed that significantly more detergent-insoluble EGFP^+^ particles were present in dissociated zebrafish expressing EGFP ataxin-3 84Q compared to non-transgenic siblings (*P*=0.0214) and EGFP ataxin-3 23Q expressing transgenic fish (*P*=0.0343, Fig. 5F). The size of detergent-insoluble EGFP^+^ particles was again similar across the analysed genotypes, with a mean detergent-insoluble particle diameter of ∼3 µm by 6 dpf (Fig. 5G).

As proof of the utility of the FloIT approach to assess the efficacy of drug treatments aimed at modifying protein aggregate formation and detergent-insoluble protein species, EGFP ataxin-3 84Q larvae were treated with 3 mM chloroquine, an autophagy inhibitor, for 24 h. A 1.5-fold increase in detergent-insoluble EGFP particles was observed in chloroquine-treated larvae compared to vehicle control larvae from the same clutch (*P*=0.012, Fig. 5H). FloIT also detected a difference in the number of detergent-insoluble EGFP^+^ particles present following 24-h treatment with the autophagy inducer calpeptin (*P*=0.006, Fig. 5I). Post-hoc comparisons revealed that treatment with 25 μM calpeptin produced a statistically significant decrease in detergent-insoluble particles compared to vehicle treatment (*P*=0.038). Treatment with 50 μM and 100 μM doses appeared to produce a decrease in detergent-insoluble particles; however, these comparisons did not reach statistical significance (*P*=0.055 and *P*=0.053, respectively).

## DISCUSSION

### A flow cytometry approach detects protein aggregates in a cell culture model of SCA3

Here, we report that neuronal-like (neuroblastoma) SHSY5Y cells expressing EGFP ataxin-3 containing a polyQ expansion (84 polyQ repeats) develop EGFP^+^ protein aggregates. These findings align with existing experimental evidence that suggests polyglutamine-expanded ataxin-3 can form protein aggregates within cultured murine or human neuroblastoma cells (Schmidt et al., 1998; Santana et al., 2020; Yoshizawa et al., 2001; van Well et al., 2019). Interestingly, we observed ataxin-3 protein aggregates predominately in the cell cytoplasm, contrasting with reports of intranuclear ataxin-3^+^ inclusions in SCA3 brain tissue (Schmidt et al., 1998; Yamada et al., 2000; Paulson et al., 1997; Rüb et al., 2008). Our findings of predominately cytoplasmic ataxin-3 protein aggregates are consistent with existing evidence of cytoplasmic protein aggregates in cultured cells transiently expressing ataxin-3 constructs with EGFP fused to ataxin-3 at the N-terminus (van Well et al., 2019; Joshi et al., 2019; Pavel et al., 2016; Khan et al., 2019; Rajamani et al., 2017; Chang et al., 2014; Upadhyay et al., 2018). In contrast, experiments utilising ataxin-3 constructs with EGFP fused to the C-terminus of the ataxin-3 protein produce ataxin-3 aggregates found within the cell nucleus (Weishäupl et al., 2019). As such, the position of the fused fluorescent protein may alter the localisation of the observed protein aggregates.

In addition to examining protein aggregation in our cell culture and transgenic zebrafish models of SCA3, we also used western blotting to examine the expression of ataxin-3 protein across our models. The purpose of our western blotting experiments was to confirm that the increased presence of EGFP ataxin-3 aggregates in our models expressing mutant expanded ataxin-3 was not due to underlying differences in protein expression. In our cell cultures, we found the expression of ataxin-3 to be significantly higher in cells transiently expressing EGFP-fused ataxin-3 with 28 polyQ repeats compared to cells transiently expressing EGFP-fused ataxin-3 with 84 polyQ repeats. In our transgenic zebrafish, expression of human ataxin-3 was similar across larvae expressing EGFP ataxin-3 23Q and EGFP ataxin-3 84Q; however, transgene expression may vary across different clutches of zebrafish embryos. This suggests that the increased presence of ataxin-3^+^ protein aggregates in SCA3 cells and transgenic zebrafish is not a consequence of underlying differences in the expression of the ataxin-3 protein, but is more likely due to pathological expansion of the polyQ region within the ataxin-3 protein, as reported previously (Haacke et al., 2006; Evers et al., 2014; Ellisdon et al., 2006; Bevivino and Loll, 2001).

Next, we aimed to determine whether we could employ the high-throughput flow cytometric analysis approach FloIT (Whiten et al., 2016) to quantify the number and size of detergent-insoluble ataxin-3 particles in SCA3 cells. This method has been previously used to quantify TDP-43, SOD1 and huntingtin^+^ inclusions (Whiten et al., 2016; McAlary et al., 2016; Zeineddine et al., 2017a,b), and other cellular processes associated with protein aggregation, including heat shock response induction (San Gil et al., 2020), the role of molecular chaperones in suppressing protein aggregation (McMahon et al., 2021) and chaperone-assisted selective autophagy (Adriaenssens et al., 2020). Here, we provide the first adaption of FloIT to examine the presence of detergent-insoluble protein species in a model of spinocerebellar ataxia. FloIT analysis revealed the presence of more Triton X-100 insoluble EGFP^+^ aggregates in cells expressing EGFP ataxin-3 84Q than those expressing EGFP ataxin-3 28Q or EGFP controls. This finding is consistent with existing reports that use western blotting and filter trap assays to provide evidence of detergent-insoluble forms of ataxin-3 in cell culture models expressing mutant expanded ataxin-3 fused to GFP (Joshi et al., 2019; Chang et al., 2014; Weishäupl et al., 2019).

In the present study, we used microscopy to determine the transfection efficiency in our cell culture models. This approach contrasts with previous publications that have used FloIT, in which flow cytometry was used to determine the transfection efficiency (Whiten et al., 2016; McAlary et al., 2016; Zeineddine et al., 2017a,b; San Gil et al., 2020). It is possible that quantification of the proportion of transfected cells using microscopy may yield a lower detection threshold than more sensitive flow cytometry methods, resulting in a lower reported transfection efficiency. As transfection efficiency is incorporated into the FloIT equation, decreased transfection efficiency could decrease the denominator in the FloIT equation and thus alter the total number of insoluble particles per 100 transfected nuclei detected. Therefore, when using FloIT, it is important to consider how the different methodological approaches used to calculate transfection efficiency could alter the overall number of insoluble particles per nuclei detected. It is recommended that future experiments remain consistent in the approach employed as inconsistency in the calculation of transfection efficiency could introduce variability.

Our flow cytometry findings are supported by existing reports of ataxin-3 aggregate formation and size, aligning with findings from Weishäupl et al. (2019) that demonstrated that cultured cells transiently transfected with human ataxin-3 constructs develop ataxin-3^+^ aggregates within 24 h. Interestingly, Weishäupl et al. (2019) did not detect a significant difference in the number of aggregates present at 24-h post transfection in cells expressing ataxin-3 with a short polyQ region and cells expressing ataxin-3 with a long polyQ region, conflicting with the present findings. However, Weishäupl et al. (2019) investigated ataxin-3 proteinopathy in an immortal human embryonic kidney cell line, whereas the present study used human neuronal-like (neuroblastoma) SHSY5Y cells. It is possible that these different cell lines may possess different intrinsic susceptibilities to aggregate formation. Additionally, it is possible that differences in protein expression could alter the amount of proteinopathy present (Kiefhaber et al., 1991; Tartaglia et al., 2007). Indeed, gene expression may also influence the formation of protein aggregates in cell culture models (Tartaglia et al., 2007). Furthermore, contrasting methodological approaches were employed, with Weishäupl et al. (2019) using microscopy to visualise and quantify ataxin-3 proteinopathy, which may lack the sensitivity required to detect ataxin-3 aggregates at this timepoint.

### Transgenic zebrafish expressing mutant ataxin-3 develop protein aggregates from 2 days of age

Here, we provide the first adaption of FloIT for use with an *in vivo* model. We were able to successfully quantify the number of Triton X-100-insoluble EGFP^+^ protein aggregates present within our transgenic zebrafish model of SCA3 that express EGFP fused to human ataxin-3 under a neuronal promotor (HuC, elavl3). We found that our transgenic zebrafish expressing EGFP fused to human ataxin-3 containing a long polyQ stretch (84 glutamine residues) displayed significantly more detergent-insoluble EGFP ataxin-3 inclusions than non-transgenic siblings or zebrafish expressing ataxin-3 with a short polyQ stretch fused to EGFP (23 glutamine residues). Interestingly, this phenotype was detectable at two different timepoints in SCA3 zebrafish development: 2 dpf, before the onset of swimming deficits, and 6 dpf, when a movement phenotype is detectable (Watchon et al., 2017). This suggests that the formation of detergent-insoluble ataxin-3 aggregates may be an early disease phenotype that may contribute to the development of neurotoxicity, neurodegeneration and motor impairment. Further, we found that treatment with a compound that we have previously demonstrated improves the swimming of these zebrafish (calpeptin) does indeed also decrease the presence of ataxin-3 protein species (Watchon et al., 2017).

**Fig. 6.**
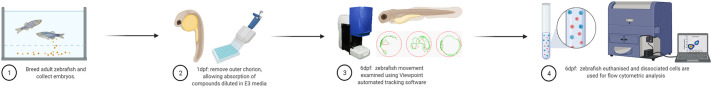
Flow cytometric analysis of cells dissociated from zebrafish larvae can be a powerful addition to high-throughput drug screening pipelines, revealing cellular level insights.

The ability to detect the presence of this protein aggregation phenotype at such an early timepoint, including the larval stages, has many advantages. First, FloIT is a relatively inexpensive and efficient analysis tool that can be used to provide a rapid readout of treatment efficacy on protein aggregation in cells dissociated from large clutches of sibling zebrafish larvae. We suggest that using FloIT to examine proteinopathy phenotypes may be valuable within high-throughput drug screening pipelines in zebrafish models of proteinopathy and neurodegenerative diseases, either on its own, or together with the tracking of locomotion behaviour (Fig. 6). Zebrafish larvae can be assayed for improvements in swimming and then dissociated into single cells at the completion of the experiment, enabling the identification of compounds that induce beneficial effects on animal movement and cellular phenotypes. At these early larval stages, drugs and small compounds can easily be dissolved in the water that the larvae are incubated within, and are absorbed by the larvae, making dosing straightforward (Zon and Peterson, 2005). Further, in comparison with more traditional methodologies for detecting proteinopathy, such as western blotting, live imaging confocal microscopy and immunostaining, this flow cytometry approach is much less time consuming and laborious. These methodologies provide information relating to the relative expression of different constructs and location of ataxin-3^+^ inclusions and thus may be useful in combination with a flow cytometry drug screening workflow to achieve the greatest insight.

To validate the use of this FloIT approach to investigate potential treatments of SCA3 and related diseases, and to confirm that the detected particles are indeed protein aggregates, we examined the effect of treatment with autophagy-modifying compounds on the number of particles detected. We hypothesised that treatment with autophagy inhibitors (3MA *in vitro* or chloroquine *in vivo*) would result in an increase in the number of insoluble EGFP ataxin-3 aggregates due to blockage of their removal. Further, we hypothesised that treatment with calpeptin, a compound known to inhibit activity of calpain proteases and induce autophagic activity, would decrease the number of detergent-insoluble EGFP ataxin-3 aggregates present. Indeed, we found that treating SHSY5Y cells or zebrafish expressing EGFP ataxin-3 84Q with autophagy inhibitor compounds increased the number of detergent-insoluble EGFP ataxin-3 aggregates detected by FloIT. In contrast, treatment with calpeptin reduced detergent-insoluble ataxin-3 particles in both cell culture and zebrafish models of SCA3. Our *in vitro* findings support existing work by Haacke et al. (2007), providing further evidence that treatment with low doses of calpeptin can decrease the presence of detergent-insoluble ataxin-3 protein aggregates in cell culture models of SCA3. Further, our zebrafish findings build upon our previous reports that calpeptin can increase activity of the autophagy protein quality control pathway in SCA3 zebrafish (Watchon et al., 2017). These findings confirm that the FloIT approach can be used to examine the effect of autophagy modulators and validates the approach for the quantification of ataxin-3 aggregates. If the approach were detecting random debris proteins or undigested whole cells, the observed fluctuations in total detergent-insoluble protein species would not have been affected by the administration of autophagy-modifying compounds.

One caveat of this approach is that the FloIT protocol requires the expression of the protein of interest fused to a fluorescent protein. Attachment of a fusion protein has the potential to alter protein dynamics or conformation, potentially rendering the protein more aggregation-prone than endogenous or native forms of the protein (Snapp, 2005). However, within this study, EGFP-fused mutant ataxin-3 protein was directly compared against EGFP-fused wild-type ataxin-3 protein, meaning that any increase in detergent-insoluble protein species can be attributed to the presence of the expanded polyQ stretch within the ataxin-3 protein rather than the fused fluorescent protein itself. Further, we have previously found in a separate series of experiments that SHSY5Y cells stably expressing human ataxin-3 with 28 or 84 glutamines develop ataxin-3^+^ protein aggregates when lacking EGFP fusion (Watchon et al., 2021). Additionally, we have also considered whether fusion of EGFP to human ataxin-3 may contribute to the swimming phenotypes observed in our transgenic zebrafish, and have found that transgenic zebrafish expressing human ataxin-3 (not fused to a fluorescent protein) under the same neuronal promoter also develop abnormal swimming phenotypes by 6 dpf (data not shown). These findings suggests that the protein aggregation phenotypes described in the present study are not caused by the fusion of EGFP to ataxin-3, but are likely caused by expansion of the polyQ region within the ataxin-3 protein. It is important to conduct similar control experiments within future studies using the FloIT approach to ensure that fusion of a fluorescent protein does not increase the aggregation propensity of the studied protein of interest.

The FloIT methodology is one of many recent flow cytometric methodologies developed to investigate protein aggregation in cultured cells expressing aggregation-prone proteins fused to fluorescent proteins. Other approaches include PulSA, a flow cytometric approach that allows the identification of intact cells containing protein inclusions or aggregates. PulSA can be used to identify cellular populations containing protein inclusions or aggregates based on differences in cell size and shape (pulse width and height) (Ramdzan et al., 2012). Similarly, cultured cells can be lysed with 0.03% saponin, allowing diffusion of soluble proteins into the extracellular space and trapping insoluble proteins (and fused fluorescent proteins) within the cell (Pokrishevsky et al., 2018). However, PulSA has been found to be ineffective at identifying cells with a low number of inclusions or aggregates and may be more suited to identifying cells containing many aggregates/inclusions per cell (Whiten et al., 2016). For this reason, FloIT may be more suitable for the rapid quantification of protein aggregates in cell populations in which many cells harbour sparse (1-3) protein aggregates. Further, PulSA or saponin approaches provide an estimation of the number of cells within a population affected by protein inclusions or aggregates; however, as FloIT lyses cell membranes this type of information cannot be obtained from this approach. Researchers should consider which approach may be most suited to their experimental aims before embarking on flow cytometric analysis of proteinopathy.

In conclusion, our findings highlight the utility of FloIT as a rapid approach to quantify protein aggregation, which can be used to screen novel compounds *in vitro* and *in vivo*. We report this novel approach of applying FloIT to transgenic zebrafish samples that can be incorporated into a drug testing pipeline to aid the identification of compounds that slow or prevent disease progression, including ameliorating protein aggregation and swimming impairment.

## MATERIALS AND METHODS

### SCA3 cell culture models

Human neuroblastoma (neuronal-like) SHSY5Y cells were grown under sterile conditions and maintained at 37°C in a sterile incubator. Cells were grown in Dulbecco's modified Eagle medium (DMEM)/Nutrient Mixture F12 Ham and supplemented with 10% fetal bovine serum and 5% CO_2_. Cells were seeded into six-well plates and allowed to grow for 1-3 days until 80-90% confluent. Once confluent, cells were transiently transfected with vectors to drive expression of EGFP fused to human ataxin-3 with a short polyQ repeat length (28Q) or human ataxin-3 with an expanded polyQ repeat length (84Q), or an EGFP only vector control. Cells were transfected with 1 μg of DNA and 1 μl of Lipofectamine 2000 (Thermo Fisher Scientific) per well and incubated for 24 h.

### Confocal microscopy of aggregates in a SHSY5Y cell model of SCA3

For confocal imaging experiments, SHSY5Y cells were seeded into a 24-well plate and grown on coverslips until reaching 60-70% confluency. Cells were then transfected with 1 μl of Lipofectamine 2000 and 1 μg of DNA per well. At 24 h post transfection, culture medium was aspirated, and cells were fixed with 4% paraformaldehyde, stained with DAPI (final concentration 14 μM) to enable identification of nuclei, then mounted using fluorescent mounting medium (Dako Omnis, Agilent, GM304). Confocal images were obtained using a Zeiss LSM-880 confocal microscope (Plan-Apochromat 40×/1.5 oil DIC M27 objective) running Zen Black software (Zeiss, Gottingen, Germany). To visualise EGFP expression, an argon laser was used. To visualise DAPI-stained nuclei, a 405 nm laser was used. A total of six *z*-stack images (13 slices spanning 6 μm) of different fields of view were acquired per coverslip and final images were obtained as maximum intensity projections using Fiji software (Schneider et al., 2012). EGFP^+^ aggregates were manually counted by a researcher blind to the experimental group. The number of aggregates per transfected cell was then calculated by averaging the results from six fields of view per coverslip.

### Preparation of SCA3 cell cultures for FloIT

For flow cytometry experiments, SHSY5Y cells were seeded into six-well plates and prepared for flow cytometry 24-h post transfection. Cultured SHSY5Y cells were imaged with a 20× objective using an EVOS microscope (Invitrogen, AMF4300) under brightfield and a GFP LED light cube to determine transfection efficiency prior to harvesting. The total number of cells and total number of GFP-expressing cells were counted using automated cell counting functions within Fiji, allowing calculation of transfection efficiency. Cells were briefly washed in PBS and harvested using 0.5% trypsin-EDTA, and then pelleted. Pelleted cells were resuspended in lysis buffer [PBS containing 0.5% Triton X-100 and complete protease inhibitors (Roche)] and DAPI was added (final concentration of 5 μM) to allow enumeration of the number of nuclei. The use of DAPI to quantify nuclei aligns with experiments by Adriaenssens et al., 2020 but contrasts with many previous reports of FloIT (Whiten et al., 2016; McAlary et al., 2016; Zeineddine et al., 2017a,b). Samples were incubated on ice and protected from light until analysis. All samples underwent flow cytometric analysis within 30 min of lysis with Triton X-100.

### Transgenic SCA3 zebrafish

The present study used a previously described zebrafish model of SCA3 (Watchon et al., 2017). Within these studies, we used embryos derived from the crossing of zebrafish driver line mq15: Tg(elavl3:Gal4-VP16; mCherry) with either line mq16: Tg(UAS:Hsa.ATXN3_23xCAG-EGFP,DsRed) or mq17: Tg(UAS:Hsa.ATXN3_84xCAG-EGFP,DsRed), resulting in transgenic zebrafish neuronally expressing both dsRED and EGFP-fused ATXN3 with either short (23Q) or long (84Q) polyQ lengths, respectively. In the present study, all breeding was performed by incrossing F1 transgenic males with F1 transgenic females expressing both driver and reporter lines with no outcrossing to wild-type zebrafish. All animal experiments were performed in accordance with the Australian code for the care and use of animals for scientific purposes (the Code) and approved by the Animal Ethics Committee of Macquarie University (2016/004 and 2017/019) and the Biosafety Committee of Macquarie University (Notifiable Low Risk Dealing: 5974). Zebrafish were housed in a standard recirculating aquarium system maintained at 28.5°C. Zebrafish embryos were screened for fluorescence (EGFP and dsRED) at 1 dpf to determine the expression of transgenic lines. Embryos were dechorionated and housed in six-well plates (15-25 larvae per well, housed at equal densities per group).

### Imaging of aggregates in a transgenic zebrafish model of SCA3

At 6 dpf, transgenic zebrafish were anaesthetised with 0.01% tricaine (Sigma-Aldrich, E10521) and embedded in 1% low-melting agarose (Sigma-Aldrich, A4018). Confocal imaging was performed using an upright Leica SP5 confocal microscope (40× water submersible objective). An argon laser (39% laser power) was used to excite EGFP. Then, *z*-stacks spanning the depth of the spinal cord (∼10 μm) were acquired and final images were obtained as maximum intensity projections using Fiji software (Schneider et al., 2012). EGFP^+^ aggregates were manually counted and the aggregate size was measured by a researcher blind to the experimental group.

### Western blotting

SHSY5Y cells transiently expressing EGFP, EGFP-fused human ataxin-3 with 28 glutamines or EGFP-fused human ataxin-3 with 84 glutamines were harvested for protein extraction and lysed using ice-cold RIPA solution containing protease inhibitors (Complete ULTRA tablets, Roche) and phosphatase inhibitors (PHOSstop tablets, Roche). Cells were incubated in RIPA solution for 20 min (with gentle shaking) before manual scraping was used to harvest cells. To extract protein from 2-dpf zebrafish larvae, zebrafish larvae were euthanised and homogenised in RIPA solution (0.5 μl per larvae), containing protease inhibitors, using a manual dounce.

Cellular debris was removed from protein homogenates via centrifugation (20 min at 19,000 ***g*** and 4°C). Protein concentration of the cleared supernatant was determined using a Pierce BCA (bicinchoninic acid) Protein Assay Kit (Thermo Fisher Scientific). Proteins were denatured by boiling at 95°C for 5 min in 1× Laemmli buffer (Bio-Rad) with 1× NuPAGE Reducing Agent (Life Technologies). Equal amounts of denatured proteins (20 μg) were loaded and separated by gel electrophoresis using 4-15% Tris-glycine gels (Bio-Rad). Separated proteins were then transferred to a PVDF membrane (0.45 μm pore size) for immunoblotting. Immunoblots were probed with primary antibodies against human ataxin-3 (produced in rabbit; a gift from H. Paulson, University of Michigan, Ann Arbor, MI, USA) and GAPDH (produced in mouse, Proteintech). Immunoblots were probed with appropriate horseradish peroxidase secondary antibodies (Promega) and visualised by chemiluminescence (SuperSignal detection kit, Pierce) using an ImageQuant LAS4000 imaging system.

### Dissociation of euthanised zebrafish larvae for flow cytometry

Dissociation of whole zebrafish was adapted from methods described previously by Acosta et al. (2018). In brief, whole zebrafish larvae (2 dpf or 6 dpf) were euthanised and larvae bodies were manually transferred to a Petri dish in a droplet of E3 medium and manually dissected into pieces (<1 mm) using a scalpel blade. Larvae pieces were then transferred to an Eppendorf tube and centrifuged (5 min, 5000 ***g***). The supernatant was removed and the pellet was enzymatically digested using 0.5% trypsin-EDTA (500 μl for 2-dpf larvae, 1000 μl for 6-dpf larvae) for 30 min at 37°C. Samples were vortexed frequently to aid digestion. Trypsinisation was stopped by the addition of 1 ml of DMEM cell culture medium containing 10% fetal bovine serum. Samples were pelleted and the DMEM/trypsin solution was removed. Samples were resuspended in 200**-**500 μl of PBS containing 0.5% Triton X-100 and 1× RedDot^TM^ far red solution (Gene Target Solutions, 40060) to identify nuclei.

In order to confirm that cells could survive the dissociation process, a subset of experiments were performed whereby dissociated cells were not used for flow cytometry and instead were stained with Hoechst 33342 live cell stain (Thermo Fisher Scientific, final concentration 400 μM) and imaged using an EVOS microscope (20× objective). The total number of cells and total number of Hoechst^+^ cells were counted using Fiji automated cell counting functions, allowing the calculation of cell survival post dissociation (Fig. S1A).

### Drug treatments in SCA3 cell cultures and transgenic SCA3 zebrafish

As a proof of principle, we examined the effect of prior treatment with compounds known to alter the activity of the autophagy protein quality control pathway on the number of detergent-insoluble ataxin-3 particles, detected using flow cytometry, in SCA3 cell cultures and transgenic zebrafish. For autophagy inhibition experiments, cultured cells were treated with 3MA (5 mM, Cayman Chemical, 13121) and zebrafish larvae were treated with chloroquine diphosphate (3 mM, Sigma-Aldrich, C6628), two commonly used inhibitors of autophagy (Klionsky et al., 2021). To examine the effect of autophagy induction on detergent-insoluble ataxin-3, calpeptin (Cayman Chemical, 14593), a compound known to induce autophagy in SCA3 zebrafish (Watchon et al., 2017) and suppress protein aggregation in cell culture (Williams et al., 2008; Haacke et al., 2007), was administered at doses ranging from 1-5 μM in cell culture and 25-100 μM for zebrafish. For drug treatments of SHSY5Y cells transiently expressing human ataxin-3, 3MA or calpeptin treatments were dissolved in culture medium (DMEM/Nutrient Mixture F12 Ham supplemented with 10% fetal bovine serum) and applied immediately before the addition of transfection reagents. For drug treatments of transgenic zebrafish, chloroquine diphosphate or calpeptin were diluted in E3 raising medium and larvae were placed in drug treatments for a minimum of 24 h. All drug treatments were dissolved in DMSO to create stock solutions. The final maximum percentage of DMSO for cell culture drug treatments reached 0.003%, whereas zebrafish experiments did not exceed a final maximum percentage of 0.01%. Stock solutions were aliquoted and stored at −20°C. Treatment with DMSO alone acted as the vehicle control and the effect of all examined drug treatments were directly compared to vehicle-treated controls.

### Flow cytometry analysis approach

Flow cytometry was performed using a BD Biosciences LSR Fortessa analytical flow cytometer running FACS DIVA software and maintained according to the manufacturer's instructions. Excitation wavelengths and emission collection windows were set as follows: DAPI (355 nm, 525/50 nm); EGFP (488 nm, 530/30 nm); and RedDot (640 nM, 670/30 nm). A minimum of 20,000 events were captured for experiments involving cultured cells and 50,000 events were captured for experiments involving dissociated cells from whole zebrafish larvae. The fluorescence of EGFP-expressing cells was compared to an untransfected or non-transgenic control sample. Nuclei were identified and quantified based on the intensity of UV/infrared fluorescence and particle size (forward scatter). The number of detergent-insoluble EGFP particles, indicating insoluble EGFP-fused ataxin-3 particles, were analysed based on GFP fluorescence intensity and forward scatter (Whiten et al., 2016). For genotype comparisons, the number of detergent-insoluble EGFP^+^ particles was determined using the FloIT equation published by Whiten et al. (2016): 100×(number of insoluble GFP particles/number of nuclei×transfection efficiency). This analysis approach allows for the comparison of detergent-insoluble particles across different transgenic expression models. For comparison of treatment effects on the same transgenic model, the number of detergent-insoluble EGFP^+^ particles per number of nuclei present was calculated and presented as a fold change relative to the vehicle-treated control group. The FSC threshold was set to 200 arbitrary units to ensure small insoluble particles were included in the analysis. Axes were set to log^10^ for all experiments. Non-fluorescent microspheres (Thermo Fisher Scientific, F13838) of a known diameter (1**-**15 μm) were used to equate forward scatter measurements (arbitrary units) to a precise micron diameter (Fig. S1B).

### Data analysis

Data analysis was performed using GraphPad Prism (Version 8) software. Group comparisons were made using one-way ANOVA, followed by a Tukey's post-hoc test to identify differences. In cases in which only two groups were compared, comparisons were made using Student's *t*-test. All graphs display group mean data±s.e.m.

## Supplementary Material

Supplementary information
