# Supplementary material for: Flow cytometry allows rapid detection of protein aggregates in cellular and zebrafish models of spinocerebellar ataxia 3
Source: Dis Model Mech. 2021 Oct 11;14(10):dmm049023. doi: 10.1242/dmm.049023 (PMC8524651; doi:10.1242/dmm.049023)
Supplement: Supplementary information [file dmm-14-049023-s1.pdf]

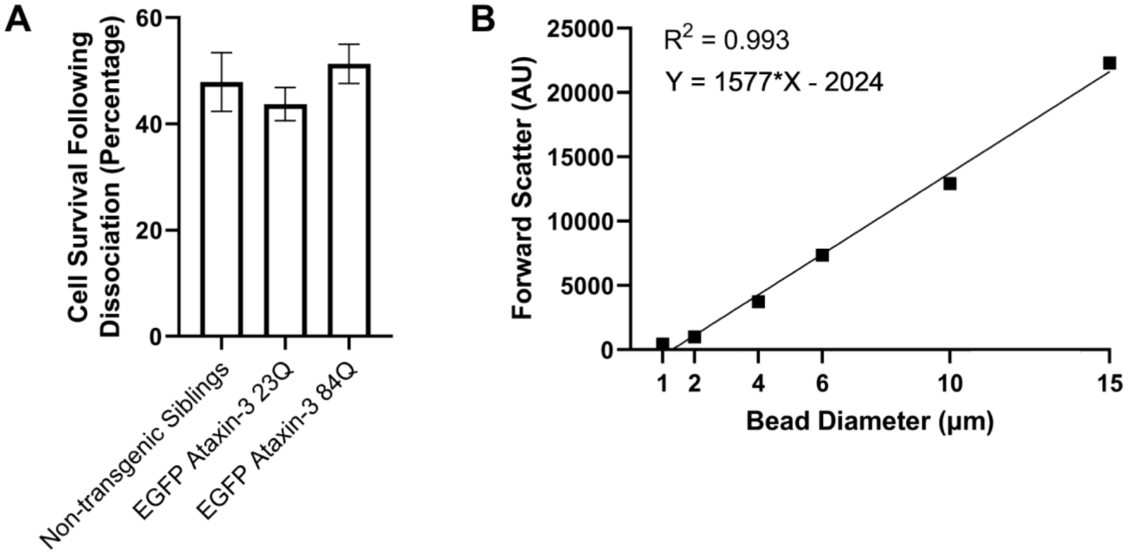

Fig. S1. (A) Staining of cells dissociated from 6 day-old zebrafish revealed greater than 40% of cells were stained with Hoechst live cell stain, suggesting greater than 40% survival post-dissociation. (B) Beads of a known micron diameter were used to calculate the relative size of insoluble GFP-positive particles using the displayed standard curve.

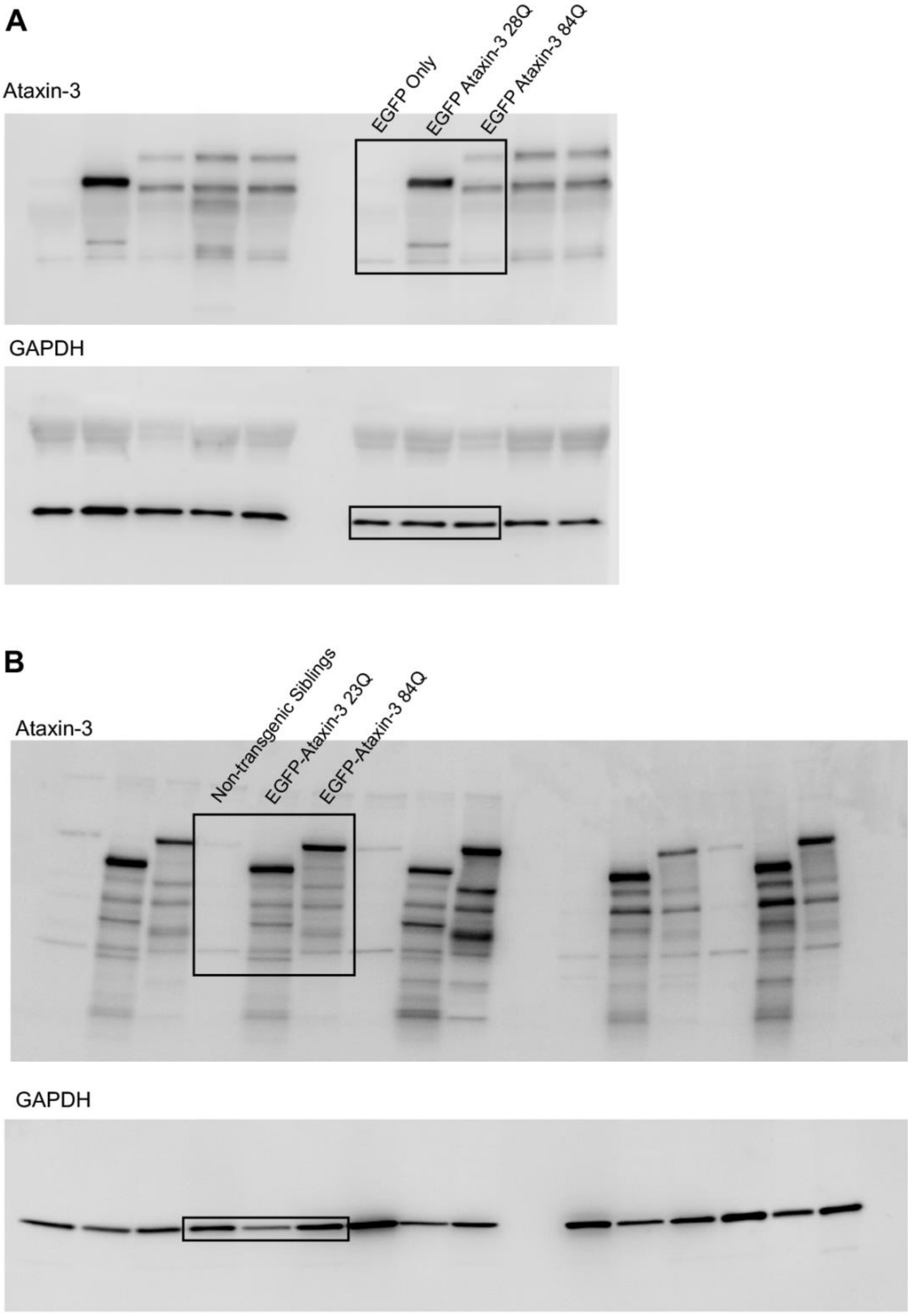

Fig. S2. (A) Uncropped immunoblot images from Figure 1. (B) Uncropped immunoblot images from Figure 4.

Disease Models & Mechanisms • Supplementary information
